# Viability Study on the Use of Three Different Gels for 3D Food Printing †

**DOI:** 10.3390/gels9090736

**Published:** 2023-09-10

**Authors:** Adrián Matas, Carmen Molina-Montero, Marta Igual, Purificación García-Segovia, Javier Martínez-Monzó

**Affiliations:** i-Food, IUIA, Food Technology Department, Universitat Politècnica de València, Camino de Vera s/n, 46022 Valencia, Spain; admagi@doctor.upv.es (A.M.); mamomon3@doctor.upv.es (C.M.-M.); marigra@upvnet.upv.es (M.I.); pugarse@tal.upv.es (P.G.-S.)

**Keywords:** 3D food printing (3DFP), rheological properties, extrusion analysis, image essay, gels

## Abstract

Three-dimensional food printing is one of the modern techniques for food customization. The difficulty of this technique lies in the formulation of new matrices. These new formulations must have good extrusion characteristics and, at the same time, maintain the structure once printed. These qualities are related to textural and rheological properties. Printability studies are those whose objective is to know the above properties. Some authors have correlated printability with rheological and physicochemical parameters. The aim of this study was to characterize three gels to test prediction models and to determine the most important rheological and textural parameters (G′, G″, Tanδ, maxF, average) in printability. The formulations studied were bovine gelatin (4%) with kappa-carrageenan (0.5%) (Gb + K), porcine gelatin (5%) plus iota-carrageenan (2%) (Gp + I), and methylcellulose (4%) (MC). The samples were characterized by an oscillatory test for the rheological properties and an extrusion test for the textural properties. In addition, the density was obtained to apply the predictive models and correlate the rheological and textural parameters to determine their influence. Gp + I and Gb + K showed higher values of maximum force in the extrusion test than MC, but MC had less deviation in the mean force during the test. All the samples showed a predominantly elastic behavior and damping factor (Tanδ) between 0.14 (Gb + K) and 0.37 (MC). It was observed that the tangent of the phase angle (Tanδ) had a large positive influence on the maximum and average force studied in the extrusion tests. The sample results did not match 100% with the predictions made from the models. It was possible to print samples that were higher in height without obtaining deformations over time of more than 5%. Further work is needed to optimize models and parameters for more accurate prediction.

## 1. Introduction

Three-dimensional food printing (3DFP) has been considered an innovative technique in recent years to develop food products. This technique has great potential both in terms of research and application in food industry purposes. It allows the personalization of food, both at the sensory and nutritional level. It offers great versatility, producing samples with different shapes, dimensions, internal structures, flavors, textures, etc. [1].

Within 3D printing, the extrusion additive deposition technique has become the most widely used. To carry out a product print, the dimensions and shapes of the product must first be designed with the help of software (Tinkercad, https://www.tinkercad.com/) (accessed on 16 July 2023) or downloaded from libraries of figures (Thingiverse, https://www.thingiverse.com/) (accessed on 16 July 2023) to obtain a stereolithography (STL) file. Afterward, this file is introduced into a slicer program (Ultimaker Cura, ver. 4.3) where the printing conditions of the figure are programmed, such as the printing speed, travel speed (when not deposited), layer height, layer type, and quantity of internal structure. With the G.code file obtained from the segmentation program, the file can be inserted into the printer to print the desired figure.

In recent years, there has been an increase in 3D printing publications, from 2099 publications between 2011 and 2015 to 19,248 from 2016 to 2020 [2]. In food science and technology, 3D printing has been applied to produce different products such as biscuits [3,4], mashed potatoes [5], fruits and vegetables [6], gels [7,8,9], cereal snacks [10,11], bread [12], chocolate [13], peanut butter, and cream cheese [7]. Three-dimensional printing has also been used a tool for by-products [3].

Despite all the food applications that have already been studied, a fundamental task is the expansion of new formulations as well as the optimization of existing ones. To achieve the aim of obtaining suitable biomaterials for correct printing, it is important to study their printability. Printability is a characteristic of inks that defines the ability of a material to be printed (extrudability) and to maintain its shape and structure after printing (buildability) and is related to rheological and textural properties [14].

There is a great diversity of behavior of different ingredients and a significant variety of food formulations to form printable doughs. This is the reason why it is such a tedious job to find those formulations that are viable for printing and keep the shape only by trial and error. For this reason, some authors have attempted to predict the stability exhibited by samples by developing models that depend on rheological and textural properties.

Some authors have pointed out that there is still a long way to go to find the rheological and textural relationships that determine the suitability of biomaterials for printing [15,16]. Other authors have attempted to narrow down some ranges of parameters that allow good food printings. In more recent studies, regarding the rheological properties of food inks, the information gathered where samples were correctly printed involved an elastic modulus (G′) and viscous modulus (G″) with values between 100 Pa and 10,000 Pa [2]. On the other hand, food materials are normally expected to exhibit pseudoplastic behavior. Pseudoplastic behavior is characterized by a decrease in the viscosity of the sample as the stress applied to the sample increases. Interestingly, biomaterials exhibit this behavior because increasing the applied stress and decreasing the viscosity allows the sample to flow through the needle properly without problems. Once the sample is printed and the effort applied to print ceases, the sample recovers its resting state and again presents a higher viscosity that allows it to maintain the given shape and structure. The range of viscosities for proper prints obtained from some studies is from 1000 Pa to 10 Pa, corresponding to shear rates from 0.1 s^−1^ to 500 s^−1^, respectively [2].

In terms of textural properties, many authors have used Texture Profile Analysis (TPA) to assess these characteristics. This technique includes a series of parameters such as fracturability, hardness, chewiness, adhesiveness, cohesiveness, gumminess, and springiness [17]. It allows us to know what a sample is like sensorially but also provides information to understand its structural stability. Hardness, resilience, cohesiveness, and springiness can indicate the structural integrity of the sample because they represent the strength and recoverability of the samples to external forces [2]. In other studies, the textural profile analysis test has been carried out to determine the existence of fracturability in the samples. Authors have discussed that the presence of fracturability in the samples is a reason to discard the sample, as fracturability causes the printed samples to be nonreproducible [18,19].

Other works have ventured into creating predictive models. These models incorporate rheological and physical parameters, such as density and height, for prediction. They can be used to obtain the deformation that samples can reach within one hour of printing [19]. All the above parameters have an impact on the accuracy of good prints and on the stability of the printed samples over time. Both terms are included in the definition of printability, mentioned above. Many researchers have studied the incorporation of ingredients to improve precision, such as cyclodextrin and chitosan [20], proteins [21], and starches [22]. To avoid covering many variables and interactions due to ingredients that incorporate the formulations of foods, the model samples chosen for this study were gels. They were composed of small percentages of hydrocolloids mixed with water. This allowed for a better focus on the mechanical properties of analyzed gels.

This study aimed to rheologically and texturally characterize different gels to apply printing behavior prediction models, extending the results presented in a previous work [23]. Then, the accuracy of the printability prediction was checked against the behavior of the samples.

## 2. Results and Discussion

### 2.1. Extrusion Test

**Figure 1:**
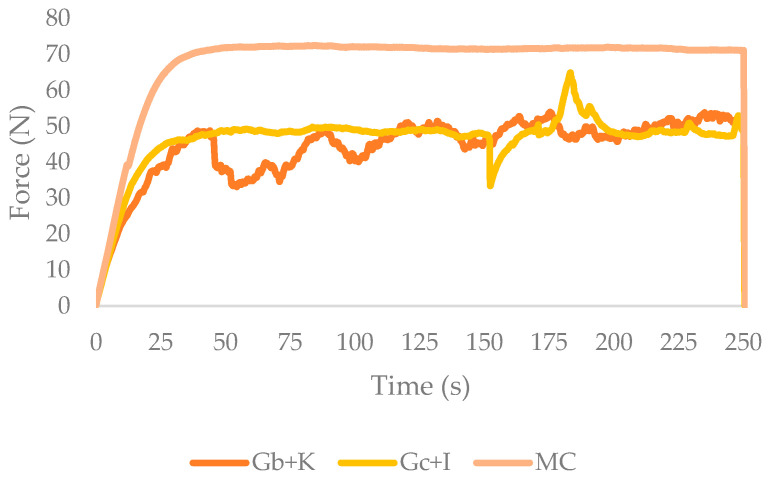
Extrusion curves for each gel (Gp + I: porcine gelatin plus iota carrageenan; Gb + K: bovine gelatin plus kappa carrageenan; MC: methylcellulose).

The extrusion test provides information on the maximum force required to print the desired sample and is related to the requirements of the printer’s extruder [5]. Figure 1 shows a representative curve of each sample in the extrusion test. With the average force, it can be deduced whether the sample shows peaks of higher or lower force, which would be attributed to particle occlusion and air bubble outgassing, respectively [24]. The presence of these peaks could also be due to the analysis of an inhomogeneous sample, poor gelation of the sample, or samples with fracturability. The gradient obtained from the results provides information on the hardness or flexibility of the sample. The higher the slope of the curve is, the greater the hardness is, and the lower the flexibility of the sample is.

The maximum force, average force, and gradient values obtained in the extrusion test for tested gels are shown in Table 1. The sample that needed the highest force to extrude was methylcellulose gel (MC), which differed little from its average force. The two gelatin samples showed a lower maximum force, with bovine gelatin plus kappa carrageenan gel (Gb + K) being the lowest, but its average force showed a greater difference concerning the maximum force. These results indicate a higher formation of air bubbles in the gelatin samples. These bubbles can be a problem when printing as they cause stops in the deposition of the material on the surface, giving figures with voids and less stability. The formation of air bubbles could be solved by controlling the agitation when making the gel or by introducing an ultrasound phase before gelation.

Looking at the gradient of the samples, MC and porcine gelatin plus iota carrageenan gel (Gp + I) did not show significant differences (*p* < 0.05), while Gb + K showed slightly lower values. These results showed that Gb + K has a more flexible behavior than the other samples.

The MC sample was the one with the highest maximum force and the lowest variation over time. In the sample Gp + I, the presence of some bubbles could be observed, as a punctual decrease in the force was observed, and a subsequent increase in the same force was observed when the air was eliminated. Gb + K had a very irregular line, which could be interpreted as a sample that presents fractures when extruded. The samples that show fracturability in the tests are usually discarded due to the poor reproducibility of the figures to be printed [18].

### 2.2. Rheological Properties

The rheological parameters provide insight into the behavior of the gels as well as the calculation of factors used for the prediction of the stability of the 3D figures [25]. Table 2 shows the results of the rheological parameters for the three gels studied. Data values at 1 Hz were used to compare the results. The results showed significant differences (*p* < 0.05) in all parameters for each gel. The gels presented different behaviors, but all of them were characterized by a more elastic behavior (G′ > G″ and G* ≈ G′).

Results have been compiled from studies in 3D food printing [2]. The rheological parameters obtained from successfully printed samples comply that G′ and G″ present values between 100 Pa and 10,000 Pa. Moreover, viscosities ranging from 1000 to 10 Pa correspond with rates of 0.1 to 500 s^−1^, respectively, and present a pseudoplastic behavior against shear stress.

**Figure 2:**
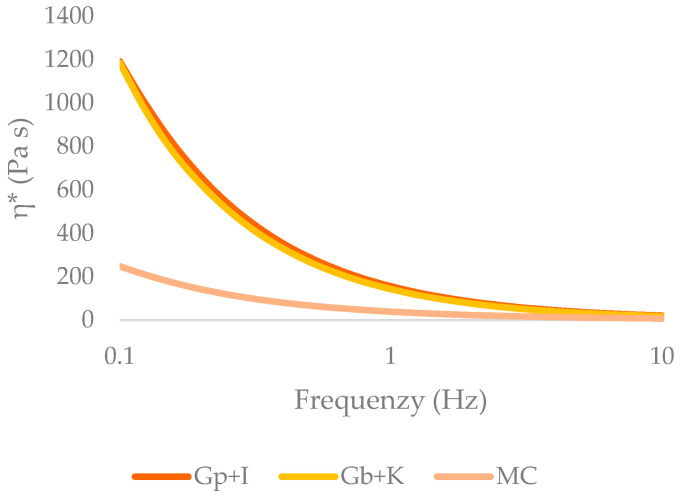
Apparent viscosity (Pa·s) versus frequency (Hz) (Gp + I: porcine gelatin plus iota carrageenan; Gb + K: bovine gelatin plus kappa carrageenan; MC: methylcellulose).

As can be seen in Table 2, all the samples showed modulus values between 100 Pa and 10,000 Pa, except for MC, whose viscous modulus value was 84 Pa, outside the lower limit. All the samples presented values of apparent viscosity within the range mentioned above. Figure 2 shows the variation of the apparent viscosity; as the frequency increased, the apparent viscosity decreased in a lower range for MC; therefore, the gels studied showed a desired pseudoplastic behavior.

**Figure 3:**
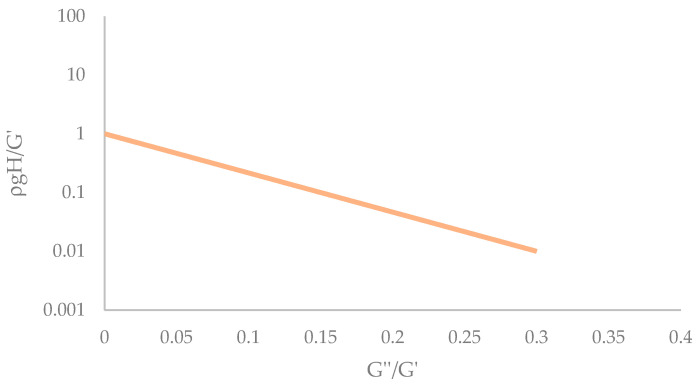
Representation of the lower curve where the samples deform 5% after 1 h resting. Curve taken from data plotted from Nidjam et al. [26].

Nidjam et al. [25] studied the relationship between different food inks with different weights (ρgH*) and rheological properties (storage modulus G′ and damping factor G″/G′) and concluded that as the storage modulus G′ increases for a given damping factor, the extent of deformation decrease [26]. These authors created a graph where two curves were represented: an upper curve, which indicated the limit of the parameters for the sample to deform by 20% after one hour of resting, and the lower one, which represented the limit at which the samples deform by 5% after one hour of resting. These two curves divided the graph into three zones depending on the deformation of the sample after 1 h of rest: <5%, 5% < x < 20%, and 20%. Focusing only on the lower curve (Figure 3), three of the points that comprised the curve could be obtained from the graph, namely 0–1, 0.15–0.1, and 0.3–0.01 of the x and y coordinates, respectively. With these points, the curve was plotted, and Equation (1) was obtained.

(1) $$\frac{\rho gH*}{G^{\prime }}=e^{-15.35\frac{G^{\prime \prime }}{G^{\prime }}}$$

where G′ and G″ are storage and loss moduli, respectively (Pa); ρ is the density of the food ink (kg/m^3^); H* is the initial height (m); and g is the gravitational constant (m/s^2^)

With the rheological parameters obtained previously, calculating the density of each sample and applying equation 1, we obtained the maximum height of the samples at which they should be printed so that after 1 h of rest, their deformation would be a maximum of 5% according to the Nidjam model [26]. The result of the maximum height at which each of the samples should be printed was 0.77 cm for G + I, 1 cm for Gb + K, and 0.008 cm for MC. With the results of the heights obtained, the most appropriate use for each of the samples could be discussed. The gelatin composite samples would allow the printing of figures up to 1 cm in height, while the MC sample would be suitable for 2D structures as a surface decoration. It should be noted that these heights were calculated for a deformation of 5% after 1 h at rest; in the case of consuming freshly printed food or undergoing post-treatment, higher heights would be allowed without affecting the result.

### 2.3. Pearson’s Correlation Coefficient

Table 3 shows Pearson’s correlation coefficients between the rheological and extrusion parameters. A correlation effect was observed between the rheological parameters and the maximum and average force except for the viscous modulus. G*, G′, and/or η* had a negative correlation; when increased, the maximum and average force decreased. On the other hand, Tanδ had the highest correlation factor; when Tanδ increased, the effect was positive, and the maximum and average force increased.

### 2.4. Image Analysis

Image analysis makes it possible to quantify the proportions of the printed figures. In this case, by measuring the height and diameter at different heights after printing and after one hour, it was possible to observe the deformation achieved.

**Figure 4:**
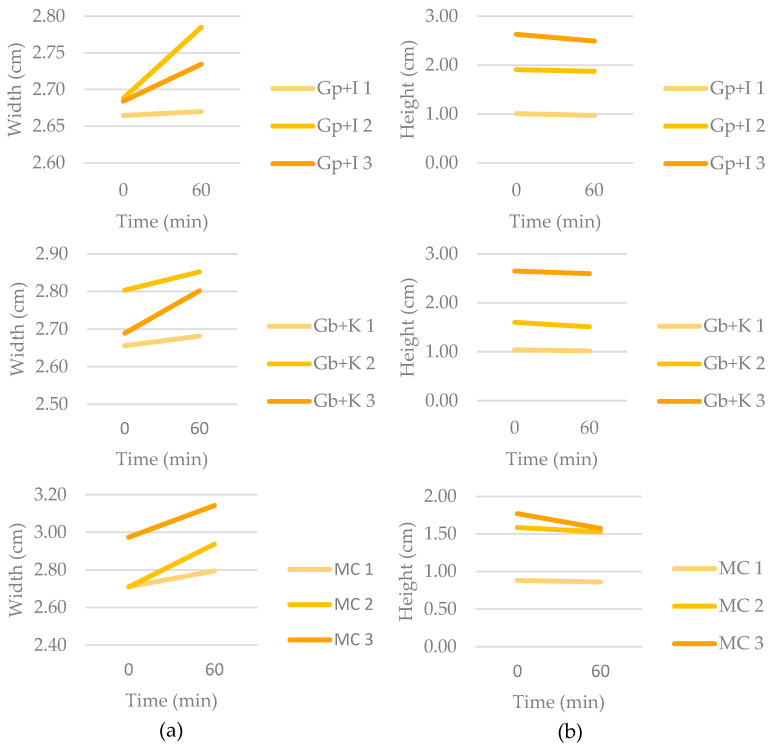
Evolution of proportions at time 0 min and 60 min: (**a**) evolution of width (diameter at different height); (**b**) evolution of height.

Figure 4 shows the evolution in the proportion of width (diameter at different heights) and height of the figures at times 0 and 60 min. A trend was observed in the evolution of the proportions of the figures. Whereas the height decreased over time, the diameter at different heights increased. This means that the samples become flatter over time because of gravity; this effect was observed in other studies [26].

The changes observed were not very drastic in the samples; in some cases, changes were by decimals, and others were practically unchanged. It should be noted that the samples were not printed in the desired proportions. Depending on the biomaterial, the initial printed figure showed proportion values closer to those designed. The Gp + I sample was the best printed initially by respecting the designed height of 1cm and 2 cm. Gb + K had only the 1 cm sample correctly printed, although the 3 cm sample had similar proportions to Gp + I 3 cm. MC presented the proportions least adjusted to the model, thus showing its limitation in creating figures in height.

**Figure 5:**
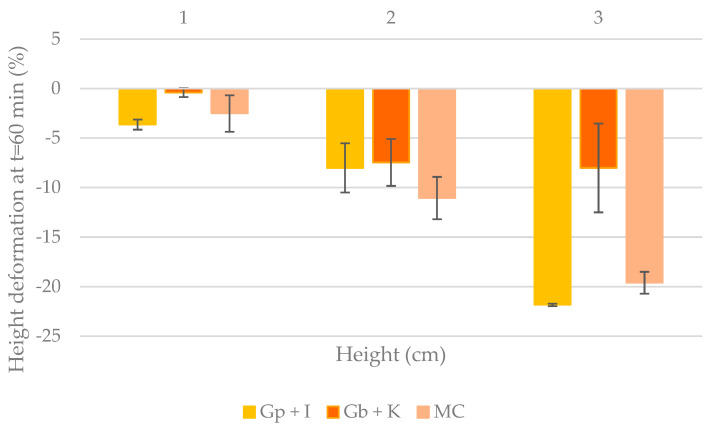
Deformation of height after 1 h of printing the samples. (Gp + I: porcine gelatin plus iota carrageenan; Gb + K: bovine gelatin plus kappa carrageenan; MC: methylcellulose).

To compare the changes in the samples with each other and with the predictions made, the height deformation was calculated. Figure 5 shows the height deformation (in percent) of the samples after 1 h.

It was observed that the 1 cm samples showed the lowest deformation percentages. None of them exceeded 5% deformation, the percentage expected by the prediction. Gb + K was the sample with the least deformation, followed by Gp + I, which had lower prediction heights of 0.77 cm and 1 cm, respectively. It should be noted that MC did not start at 1 cm like the previous samples; it deformed while printing, so its deformation was higher. The deformation of MC 1 cm would take values higher than 5% since its predicted maximum height to reach this percentage was 0.008 cm.

In figures with 2 cm and 3 cm of height, it was observed that those with the lowest deformation percentages were Gb + K followed by Gp + I at 2 cm and MC at 3 cm. As previously mentioned, freshly printed MC has lower heights than the designated ones because, during printing, the sample starts to flatten as it does not support its weight.

## 3. Conclusions

In this study, three samples of gels composed of porcine gelatin plus iota carrageenan, bovine gelatin plus kappa carrageenan, and methylcellulose were characterized rheologically and texturally.

With the predictive model and the data obtained previously, the maximum height of the samples, which deformed by 5% at most after one hour of printing, was obtained. These heights were 1, 0.77, and 0.008 cm for Gp + I, Gb + K, and MC, respectively.

Image analysis showed that the predictive model would not match the results with 100% similarity. It was possible to obtain figures of 1 cm which had a deformation over time of less than 5%. Further work is needed to optimize models and parameters that predict the good or bad behaviour of the matrices.

## 4. Materials and Methods

The materials chosen were bovine gelatin, porcine gelatin, and methylcellulose. All the materials were purchased from the Sosa brand (SOSA INGREDIENTS, SLU, Navarcles, Spain). Water food colorant from Vehiné (McCormick España S.A, Sabadell, Spain) was purchased from the supermarket.

### 4.1. Gel Production

To produce the gels, different hydrocolloids were used, namely porcine gelatin (Gp), bovine gelatin (Gb), iota-carrageenan (I), kappa-carrageenan (K), and methylcellulose (MC) (Sosa, Barcelona, Spain).

Three gels were prepared: bovine gelatin plus kappa-carrageenan (Gb + K), porcine gelatin plus iota-carrageenan (Gp + I), and methylcellulose (MC).

#### 4.1.1. Bovine Gelatin Plus Kappa-Carrageenan (Gb + K)

A solution of 4% Gb and 0.5% K was prepared in distilled water. The total volume of water was separated into two equal parts; one part was heated to 65 °C to dissolve the gelatin, and the other one to 75 °C for the K solution. A colorant was added to improve the visualization of the gel. This mixture was placed in a syringe at room temperature for 1 h and then placed in a fridge at 4 °C for 30 min. The sample was allowed to warm before use at room temperature.

#### 4.1.2. Porcine Gelatin Plus Iota-Carrageenan (Gp + I)

A total of 5% of Gp and 2% of I were dissolved in distilled water. For this purpose, half of the water was heated to 65 °C and dissolved the gelatin, and the other fraction was heated to 75 °C, at which the iota-carrageenan was dissolved. A colorant was added to improve the visualization of the gel. The mixture was placed in a syringe and cooled at room temperature for 1 h 30 min.

#### 4.1.3. Methylcellulose (MC)

Next, 4% of methylcellulose was dissolved in distilled water at room temperature. Once dissolved, a colorant was added to improve the visualization of the gel; then, the dispersion was placed in a syringe and refrigerated for 24 h at 4 °C. The sample was tempered at room temperature before use.

### 4.2. Rheological Properties

The rheological properties of the three gels were characterized on a Kinexus Pro + rotational rheometer (Malvern Instruments, Worcestershire, UK) with space software at 25 °C using a stainless-steel 40 mm parallel plate geometry with a 1 mm gap. An amplitude sweep was performed to determine the linear viscoelastic region. The initial shear stress ranged from 0.1% to 100% at the end at 1 Hz frequency. The samples had a yield stress of 31.73, 101.4, and 20.01 Pa for Gp + I, MC, and Gb + K, respectively. An oscillatory test was carried out at a fixed strain of 1 Pa and a frequency range from 0.1 to 10 Hz. Complex modulus (G*), elastic modulus (G′), viscous modulus (G″), the damping factor or tan δ (G″/G′), and apparent viscosity (η*) values were obtained for different frequency values (Hz). All tests were performed in triplicate.

### 4.3. Extrusion Test

**Figure 6:**
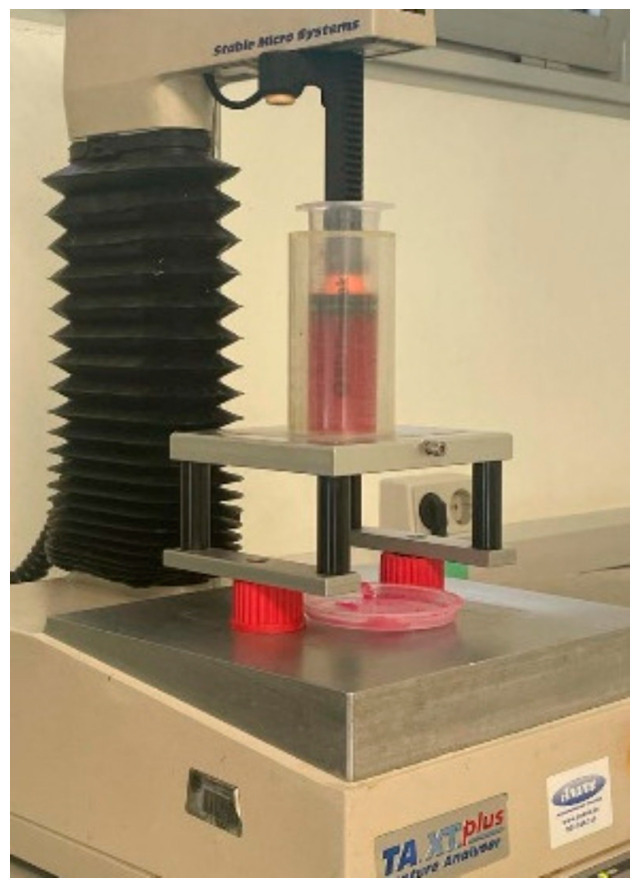
Extrusion test setup.

For the extrusion test, the printing devices and conditions were transferred (velocity, temperature, and needle diameter and syringe) (Figure 6). An extrusion test was performed using a TA.XT.plus texturometer (Stable Micro Systems, Godalming, Surrey, UK) and Texture Exponent 32 program (Stable Micro Systems, Godalming, Surrey, UK). The printer plunger, syringe (35 mm diameter) with the sample and needle (0.7 mm diameter), and a cylindrical press attachment were used for syringe stability, as Figure 6 shows. The test conditions were a 0.04 mm/s downstroke speed and 15 mm distance moved. All tests were performed at least six times.

### 4.4. Sample Printing

Samples were printed in the form of a cylinder that was 3 cm in diameter and 1, 2, and 3 cm in height. This figure was designed in Tinkercad (Tinkercad, Autodesk, Inc., San Rafael, CA, USA) and transferred to the Slicer (Alessandro Ranellucci) program. In Slicer, the printing was configured with the following parameters: needle speed, 20 mm/s; layer height, 1.7 mm; and 100% straight filling.

To produce a 3D food printer (BCN 3D+, BCN3D Technologies, Barcelona, Spain) equipped with a pasta extruder nozzle designed for food materials (BCN3D Technologies, Barcelona, Spain), the 3D printing system consisted of an extrusion system (syringe) and an X-Y-Z positioning system using stepper motors. Printing was performed at room temperature with a nozzle diameter of 0.7 mm.

### 4.5. Image Analysis

**Figure 7:**
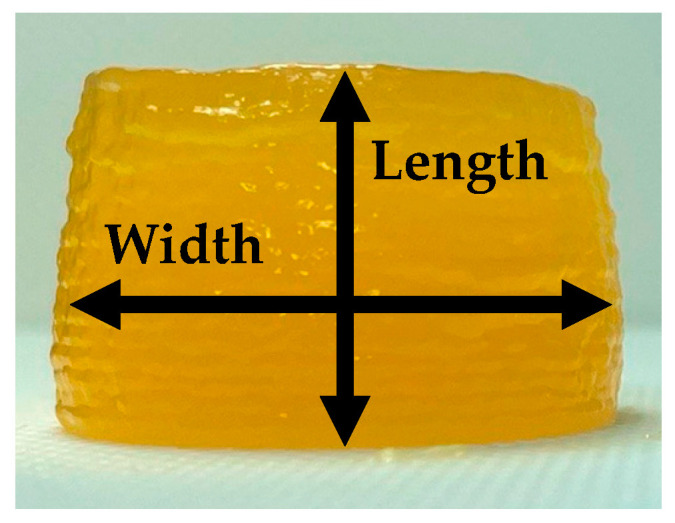
Sample data acquisition in image analysis.

Pictures of each gel’s frontal view were taken after printing and 1 h later. These images were processed in the ImageJ program (ImageJ, NIH, Washington, DC, USA) to determine the shape evolution after 1 h. From the frontal view, the width (diameter at different heights) and height were measured (Figure 7). As a metric of variation of each dimension, differences between time 0 and 1 h after figures were calculated.

### 4.6. Statistical Analysis

All tests were carried out in triplicate. Data obtained from the trials were processed with the statistical software Statgraphics Centurion 18 program, version 18.1.13 (Statgraphics Technologies, Inc., The Plains, VA, USA). An analysis of variance (ANOVA) for a 95% confidence interval (*p* < 0.05) was performed to evaluate the differences between the different samples. In addition, Pearson’s correlation coefficient between rheological and extrusion parameters with a 95% significance level was carried out.

## Figures and Tables

**Table 1:** Extrusion parameters of gels (Gp + I: porcine gelatin plus iota carrageenan; Gb + K: bovine gelatin plus kappa carrageenan; MC: methylcellulose).

Sample	Gradient (N/s)	Maximum Force (N)	Force Mean (N)
Gp + I	2.7 (0.3) ^b^	60 (11) ^b^	56 (11) ^b^
Gb + K	2.1 (0.4) ^a^	45 (9) ^a^	38 (9) ^a^
MC	2.9 (0.4) ^b^	83 (6) ^c^	82 (5) ^c^

The letters ^(a–c)^ in columns indicate the homogeneous groups between the same sample parts according to ANOVA (*p* < 0.05).

**Table 2:** Rheological parameters of gels (Gp + I: porcine gelatin plus iota carrageenan; Gb + K: bovine gelatin plus kappa carrageenan; MC: methylcellulose).

Sample	G* (Pa)	G′ (Pa)	G″ (Pa)	Tanδ	η* (Pa s)
Gp + I	978 (58) ^c^	964 (59) ^c^	159 (4) ^c^	0.165 (0.007) ^b^	156 (9) ^c^
Gb + K	894 (15) ^b^	885 (15) ^b^	125 (2) ^b^	0.1418 (0.0007) ^a^	142 (2) ^b^
MC	244 (17) ^a^	229 (17) ^a^	84 (5) ^a^	0.367 (0.005) ^c^	39 (3) ^a^

The letters ^(a–c)^ in columns indicate the homogeneous groups between the same sample parts according to ANOVA (*p* < 0.05).

**Table 3:** Pearson’s correlation coefficient between rheological and extrusion parameters.

Rheological Parameter	Gradient (N/s)	Maximum Force (N)	Average Force (N)
G* (Pa)	−0.5592	−0.7189 *	−0.7442 *
G′ (Pa)	−0.5619	−0.7226 *	−0.7476 *
G″ (Pa)	−0.3546	−0.4474	−0.4847
Tanδ (°)	0.6566	0.8265 *	0.8296 *
η* (Pa s)	−0.5592	−0.7190 *	−0.7442 *

* Correlation is significant at 0.05.

## Data Availability

Not applicable.
